# Cannabidiol Is Associated with Improved Survival in Pancreatic Cancer and Modulation of Bile Acids and Gut Microbiota

**DOI:** 10.3390/ijms26167733

**Published:** 2025-08-10

**Authors:** Pratibha Malhotra, Ranjith Palanisamy, Arunima Panda, Ilaria Casari, Janina E. E. Tirnitz-Parker, Fergal O’Gara, Robert Trengove, Krish Ragunath, Jose A. Caparros-Martin, Marco Falasca

**Affiliations:** 1 Curtin Medical School, Curtin Medical Research Institute, Curtin University, Perth, WA 6845, Australia; pratibha11malhotra@gmail.com (P.M.); ranjith@progenispharma.com (R.P.); arunima.panda@curtin.edu.au (A.P.); ilacasari@gmail.com (I.C.); n.tirnitz-parker@curtin.edu.au (J.E.E.T.-P.); robert.trengove@curtin.edu.au (R.T.); krish.ragunath@curtin.edu.au (K.R.); 2 UWA Medical School, The University of Western Australia, Perth, WA 6009, Australia; f.ogara@ucc.ie (F.O.); joseantonio.caparrosmartin@uwa.edu.au (J.A.C.-M.); 3 BIOMERIT Research Centre, School of Microbiology, University College Cork, T12 K8AF Cork, Ireland; 4 Department of Medicine and Surgery, University of Parma, Via Volturno 39, 43125 Parma, Italy

**Keywords:** pancreatic ductal adenocarcinoma, cannabinoids, gut microbiota, bile acid metabolism, cannabidiol, delta-9-tetrahydrocannabinol, biomarkers

## Abstract

Pancreatic ductal adenocarcinoma (PDAC) is among the most aggressive malignancies, with dismal survival rates. Cannabinoids have shown anticancer properties in various cancers, including PDAC. This study aimed to evaluate the anticancer effects of cannabinoids, individually and in combination, and to elucidate their mechanisms of action in a murine PDAC model (KPC mice, KRASWT/G12D/TP53WT/R172H/Pdx1-Cre+/+) that mimics human disease. Additionally, the study explored the potential link between cannabinoid action, gut microbiota modulation, and bile acid (BA) metabolism. PDAC cell lines and KPC mice were treated with delta-9-tetrahydrocannabinol (THC) and cannabidiol (CBD), either as monotherapy or in combination. Faecal pellets, caecal contents, plasma, and tissues were collected at the survival endpoint for analysis. BA profiling was performed using mass spectrometry, and the faecal microbiota was characterised by sequencing the V3-V4 region of the 16S rRNA gene. While CBD and THC synergistically reduced cell viability in PDAC cell lines, only CBD monotherapy improved survival in KPC mice. Extended survival with CBD was accompanied by changes in gut microbiota composition and BA metabolism, suggesting a possible association. Notably, the effects of CBD were different from those observed with THC alone or in combination with CBD. The study highlights a distinct role for CBD in altering BA profiles, suggesting these changes may predict responses to cannabidiol in PDAC models. Furthermore, the findings propose that targeting BA metabolism could offer a novel therapeutic strategy for PDAC.

## 1. Introduction

Pancreatic cancer is an aggressive malignancy with a five-year survival rate of approximately 13% [1]. It is the third leading cause of cancer-related deaths in the U.S., and global incidence rates are projected to increase over the next decade [2]. Pancreatic ductal adenocarcinoma (PDAC), the most common subtype, accounts for over 85% of pancreatic cancer cases [3,4]. Current treatment options for PDAC primarily involve surgery and chemotherapy [5]. Over the past decade, outcomes have improved for patients with surgically resectable PDAC [6]. However, while chemotherapy benefits some patients, its effectiveness is ultimately limited by the development of chemoresistance, a hallmark of PDAC [7]. This resistance is largely driven by the desmoplastic tumour microenvironment, a key characteristic of PDAC that fosters cancer progression, enhances metastatic potential, and reduces therapeutic efficacy [8,9,10]. Given these challenges, there is an urgent need to identify and develop novel therapeutic strategies to improve survival outcomes for PDAC patients. The anticancer potential of cannabinoids has been explored in various cancers, including those of the brain, lung, and pancreas [11]. Studies have shown that combining cannabinoids with standard chemotherapy regimens can enhance cancer cell sensitivity, thereby increasing treatment efficacy [12]. Previous work from our group demonstrated that CBD inhibits cell growth and increased sensitivity to gemcitabine in PDAC cell lines [13]. Furthermore, CBD potentiated the chemotherapeutic response of gemcitabine in a transgenic PDAC mouse model of PDAC (KPC) [13]. Growing evidence suggests that cannabis and cannabinoids influence the microbiome [14].

The involvement of gut microbiota in PDAC has also been suggested. Altered microbial profiles in the oral cavity [15,16], gut [17], duodenum [18,19], and faeces [20,21] have been associated with PDAC risk. A recent study investigated faecal microbiota alterations as potential diagnostic markers in Spanish and German PDAC cohorts [20]. The study revealed distinct microbial composition in PDAC patients compared to those with chronic pancreatitis and controls. While the authors did not investigate the mechanistic links between intestinal microbial alterations and PDAC development, reduced levels of certain microbial taxa were associated with intestinal inflammation [20,21]. Gut microbiota also contributes to tumour development and drug resistance [22,23], for instance, specific bacterial classes like *Gammaproteobacteria* influence pharmacokinetics and pharmacodynamics [24]. Beyond compositional changes, gut microbiota-associated metabolites such as bile acids (BA), have also been linked to PDAC progression [25,26]. BA are cholesterol-derived amphipathic molecules synthesised in the liver and secreted into the small intestine to facilitate lipid and vitamin absorption. Beyond their role as fat emulsifiers, BA possess endocrine and immunomodulatory functions [27,28,29]. The gut microbiome plays a crucial role in maintaining BA homeostasis by converting primary BA, such as cholic acid and chenodeoxycholic acid, into secondary BA [30]. Due to their antimicrobial properties, the composition of the BA pool reciprocally shapes the gut microbial community. BA are implicated in pancreatic injury and pancreatitis and are known to promote PDAC progression [31,32,33]. Evidence suggests that BA may contribute to gemcitabine resistance in PDAC [34]. However, there is no consensus on how the BA and gut microbiota profiles change during PDAC development. In this study, we utilised preclinical models of PDAC to assess the therapeutic potential of cannabinoid treatments. We further examined the effects of cannabinoids on gut microbiota composition and BA profiles. Our objective was to explore whether the observed therapeutic benefits of cannabinoid treatment might be associated with modulation of gut microbiota and its metabolites in PDAC models.

## 2. Results

### 2.1. CBD and THC, in Combination, Decrease Pancreatic Cancer Cell Viability

**Figure 1:**
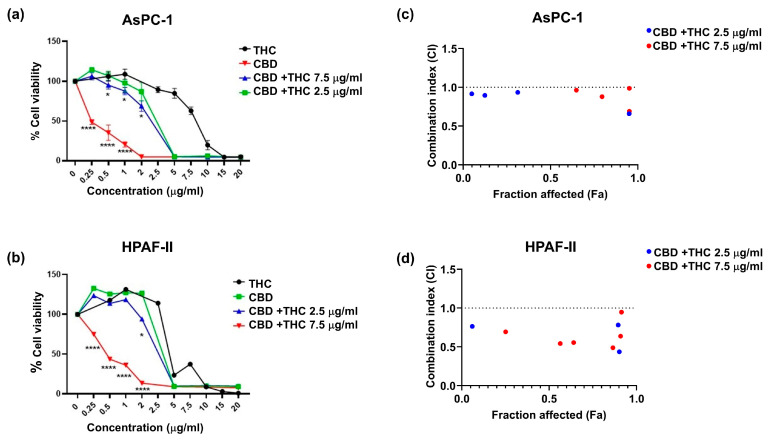
The plots depict the percentage cell viability for (**a**) AsPC-1 and (**b**) HPAF-II. The graph presents cell viability normalised to control for cannabidiol (CBD) and delta-9-tetrahydrocannabinol (THC) dose response alone and in combination. The results are presented as mean ± SEM of 3 independent experiments (n = 3). The data analysis was performed using GraphPad Prism V10.0 and CompuSyn software 1.0. The one-way analysis of variance (ANOVA) was used as the statistical test. * *p*-value < 0.05, **** *p*-value < 0.0001. Combination index plots for increasing concentrations of CBD in combination with either 2.5 μg/mL or 7.5 μg/mL of THC for AsPC-1 (**c**) and HPAF-II (**d**). Combination index (CI) vs. fraction affected (Fa) plots of effect generated for (**c**) AsPC-1 and (**d**) HPAF-II using the Chou Talay Method. The data is presented for the mean of three independent experiments. CI > 1, CI < 1, and CI = 1 represent antagonism, synergism, and no effect, respectively. This graph illustrates CI values lower than 1.

We assessed the anticancer effects of CBD and THC in AsPC-1 (Figure 1a) and HPAF-II (Figure 1b) cell lines using the MTT assay. We observed that CBD was more potent than THC in decreasing cell viability in both cell lines when treatments were administered independently, particularly at concentrations of 2.5–5 μg/mL. Based on the half-maximal inhibitory concentration (IC_50_) values for THC (HPAF-II, 8 μg/mL; AsPC-1, 6.5 μg/mL), increasing doses of CBD were combined with either 2.5 μg/mL or 7.5 μg/mL of THC for treatment. We observed a significant reduction in viability in AsPC-1 when both CBD (0.5–2 µg/mL) and THC (2.5 μg/mL) were co-administered (Figure 1a,b). On the other hand, a significant viability reduction in HPAF-II was only observed at 2 μg/mL CBD. The use of THC 7.5 μg/mL in combination with CBD demonstrated higher cytotoxicity in both cell lines than other treatments, with a significant reduction in cell viability observed at CBD 0.25–1 μg/mL in AsPC-1 and 0.25–2 μg/mL in HPAF-II cells. We determined synergistic and antagonistic effects of both CBD and THC using the CompuSyn software 1.0. Figure 1c,d presents plots of CI values (indicating synergism) for the fraction affected effect at increasing CBD concentrations combined with either 2.5 μg/mL or 7.5 μg/mL of THC in AsPC-1 cells (Figure 1c) and HPAF-II cells (Figure 1d). The plots indicate that THC 2.5 μg/mL and 7.5 μg/mL concentrations showed synergism (CI < 1) in both cell lines. In AsPC-1, THC at both concentrations produced a synergistic effect when combined with CBD at concentrations between 0.5 µg/mL and 5 µg/mL. In HPAF-II, THC 2.5 µM was synergistic when used with CBD at concentrations between 2 µg/mL and 10 µg/mL. However, THC 7.5 µg/mL in the same cell line showed synergism when combined with CBD at concentrations between 0.25 µg/mL and 10 µg/mL. Overall, the in vitro studies showed that, when combined, THC and CBD synergise and are more potent in reducing cell viability in PDAC cell lines than when used independently.

### 2.2. CBD Independently Increases Survival in the KPC Mice

Next, we investigated the effect of cannabinoids on survival outcomes in the KPC model. Once the tumour was palpable, KPC mice were intraperitoneally (i.p.) administered CBD (100 mg/kg; n = 16), THC (10 mg/kg; n = 16), CBD (100 mg/kg) +THC (10 mg/kg) (n = 17) or vehicle (n = 18). The i.p. method was chosen for treatment delivery because it provides high drug concentration and a longer half-life in the peritoneal cavity [35].

**Figure 2:**
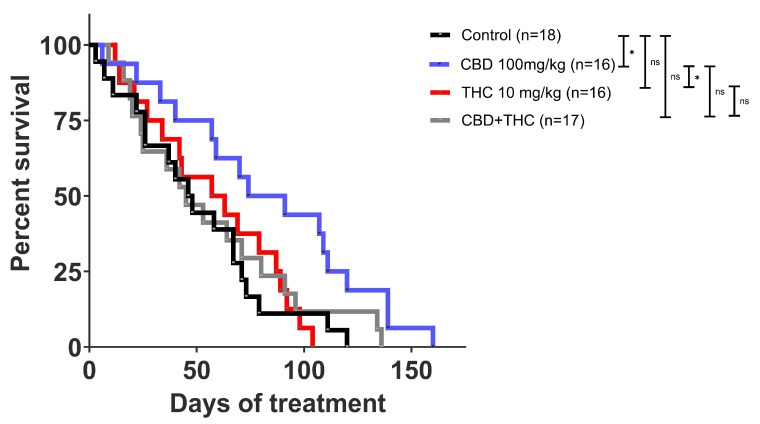
Kaplan Meier survival curve of KPC mice. Cannabidiol (CBD) treatment alone showed an improvement in survival compared to other treatments. Survival comparison of CBD (blue) compared to vehicle control (*p*-value: 0.0216), CBD compared to CBD + THC (*p*-value: 0.0560), and CBD compared to THC (*p*-value: 0.0171) treatment. The *p*-values were obtained using the Log-rank (Mantle-Cox) test (GraphPad Prism V10.0 was used for data analysis). * *p*-value < 0.05, ns—not significant.

In stark contrast to the in vitro model, we observed that CBD administration alone significantly increased the survival of KPC mice (mean 83.56 days, median 82.50, versus 50.72 mean days, 47 median days in the control group) (Figure 2 and Supplementary Table S1). Unlike our in vitro observations, THC did not potentiate the anticancer effect of CBD in vivo. Instead, THC treatment appeared to suppress the positive effects of CBD (Figure 2 and Supplementary Table S1).

### 2.3. Intraperitoneal Cannabinoid Treatments Alter the Gut Microbiota in KPC Mice

**Figure 3:**
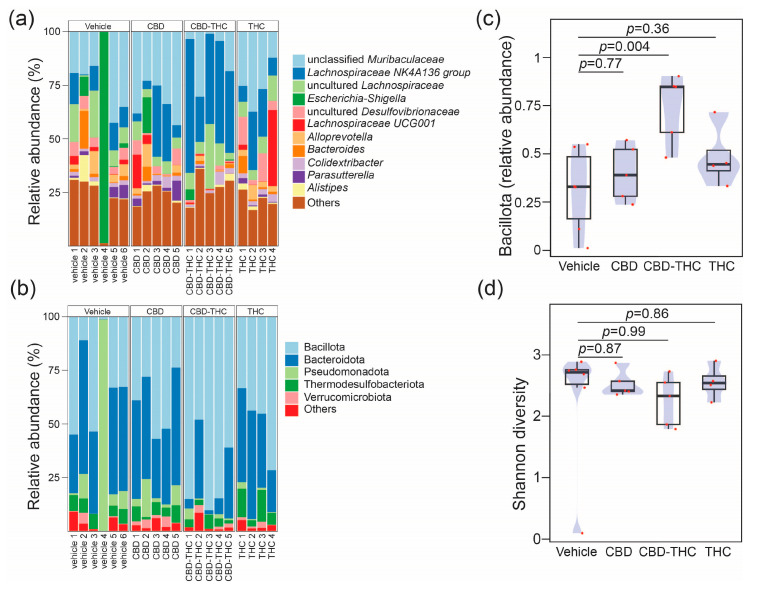
Stacked bar plots showing the relative abundance across samples resolved at the (**a**) genus level for the top 11 genus and at (**b**) phylum level. The barplot shows the relative proportions of the top 5 phyla. Each bar represents the bacterial component of the caecal microbiota of a single animal. Profiles are grouped by treatment, vehicle (define) (n = 6), CBD (n = 5), THC (n = 4), CBD + THC (n = 5). Plots representing (**c**) compositional changes in phylum Bacillota (former Firmicutes) and (**d**) Shannon diversity across the cross-sectional study. In panels (**c**,**d**) groups were compared using one-way analysis of variance (ANOVA) with Dunnett’s post hoc correction. The boxplots were overlayed with density plots (blue). The red dots represent individual samples.

**Figure 4:**
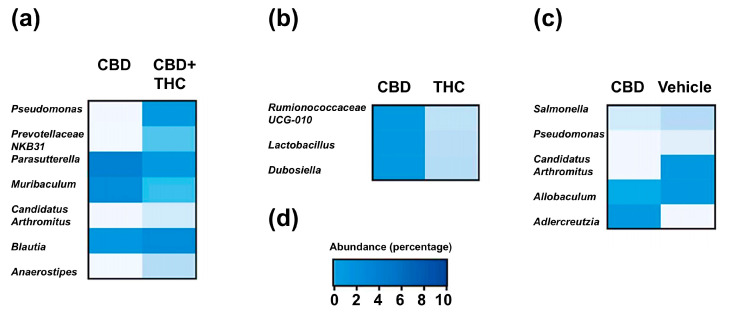
Heatmaps represent the mean relative of differentially abundant operational taxonomic units (OTUs) in comparison between treatment groups: (**a**) CBD versus CBD + THC, (**b**) CBD versus THC, and (**c**) CBD versus non-treated (vehicle) in KPC mice. Differential abundance was assessed using the ANCOM-BC algorithm, which fits a bias-corrected log-linear model to estimate log fold changes for each taxon, followed by a Wald test with FDR correction to assess statistical significance. Only taxa with FDR-adjusted *p*-value < 0.05 are shown. Rows represent individual OTUs, and columns represent treatment groups. (**d**) Colour legend for heatmaps in a, b and c represents the relative abundance (percentage).

Given the association of gut microbiota with heterogeneous responses in PDAC [36] and alterations in drug bioavailability [37], we next investigated whether modulation of the gut microbiota in KPC mice could explain the contrasting effects on survival of CBD- and THC-treated KPC animals. The i.p. administration of drugs eliminates the influence of the microbiota on the drug’s pharmacokinetics (e.g., by preventing microbial modification of the compound). This approach also enables the assessment of the gut microbiota’s contribution to the therapeutic effects of CBD and THC, removing any confounding effects related to the treatments’ direct toxicity. We used a 16S rRNA amplicon sequencing approach to profile the caecum-associated microbiota in our preclinical mouse model. The more abundant feature in the dataset was represented by an operational taxonomic unit (OTU) assigned to an unclassified member of the Family Muribaculaceae (mean relative abundance 0.23, standard deviation (SD) 0.14) (Figure 3a). At the phylum level, the microbial profiles were dominated by Bacillota (former Firmicutes) (mean relative abundance 0.47, SD 0.24) (Figure 3b). The relative proportion of microbial phyla was comparable across treatment groups except for the phylum Bacillota, which, compared to the control group, was increased after CBD-THC combined treatment (mean difference [95% family-wise confidence level] 0.43 [0.14–0.72], *p*-value after Dunnett’s test post hoc correction 0.004) (Figure 3c). Treatment with cannabinoids did not alter bacterial alpha diversity in the caecum (Figure 3d). We, however, observed a significant effect of treatment on the overall structure of the gut microbial communities, with 30% of the variance in the bacterial composition of the dataset being explained by treatment (PERMANOVA, pseudo-F: 2.27, R^2^ 0.30, *p* = 0.006). To better understand the effect of the different cannabinoids on the composition of the gut microbiota, we performed a differential abundance analysis using the ANCOM-BC algorithm [38] (Figure 4). Compared to the control group, treatment with CBD demonstrated a decrease in OTUs associated with the opportunistic pathogens *Salmonella* and *Pseudomonas*, while the extent of this effect was modest. Compared to the CBD + THC treatment, CBD treatment was associated with a lower proportion of an OTU assigned to the *Pseudomonas* taxon. CBD and CBD-THC therapies led to differential regulation of OTUs assigned to gut commensals such as *Muribaculum*, *Blautia* or *Anaerostipes* (Figure 4a). Compared to THC, CBD-treated mice showed enrichment in OTUs assigned to commensal taxa, including *Ruminococcaceae*, *Lactobacillus* and *Dubosiella* (Figure 4b). Similarly, CBD treatment was associated with increased abundance of OTUs associated with commensal bacteria assigned to the *Allobaculum* and *Adlercreutzia* taxa when compared to the microbiota of non-treated animals (Figure 4c).

### 2.4. Extended Survival Linked to Treatment with CBD Is Associated with a Reduction in the Levels of Bile Acids

**Figure 5:**
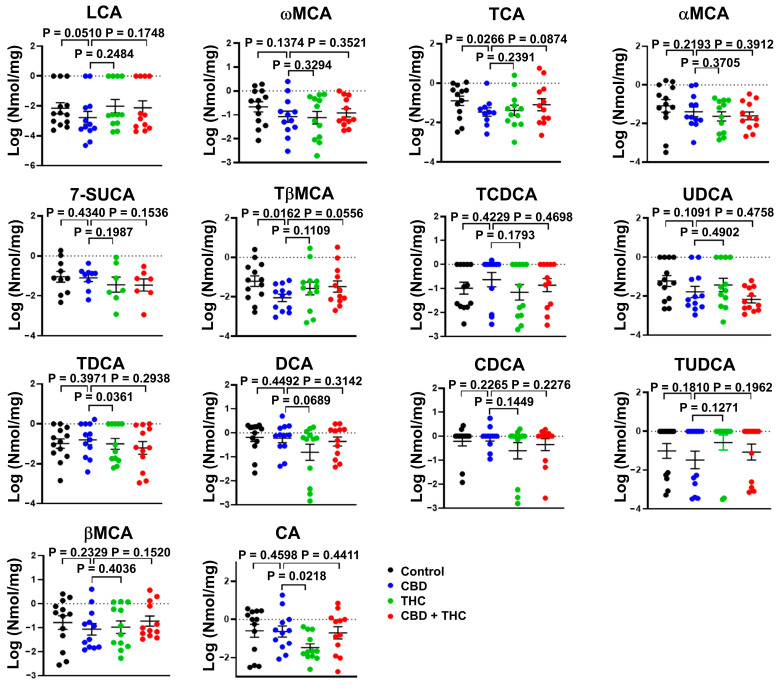
Bar plots showing the concentration of the indicated bile acids (BA) species in faeces of cannabinoid treated KPC mice. The data were log-transformed and presented as mean ± SEM. The data analysis was performed using GraphPad Prism. The Mann–Whitney test was used to evaluate the differences in the BA profile within treatment groups. bMCA, β-muricholic acid; aMCA, α-muricholic acid; wMCA, ω-muricholic acid; 7-SUCA, 7-sulphocholic acid; CA, cholic acid; DCA, deoxycholic; CDCA, chenodeoxycholic acid; LCA, lithocholic acid; TCA, taurocholic acid; TDCA, taurodeoxycholic acid; TCDCA, taurochenodeoxycholic acid; TUDCA, tauroursodeoxycholic acid; TβMCA, tauro-β-muricholic acid; UDCA, ursodeoxycholic acid.

Next, we evaluated if the benefits associated with CBD are related to the modulation of BA metabolism (Figure 5). Treatment with CBD, but not with THC or the combination of CBD and THC, specifically reduced the faecal concentration of the conjugated primary BA taurobetamuricholic acid (TβMCA, vehicle versus CBD, *p* = 0.0162) and taurocholic acid (TCA, vehicle versus CBD, *p* = 0.0266) (Figure 5). On the other hand, the faecal concentration of either total BA (Supplementary Figure S1) or other BA species were not affected by the treatments with cannabinoids (Figure 5).

**Figure 6:**
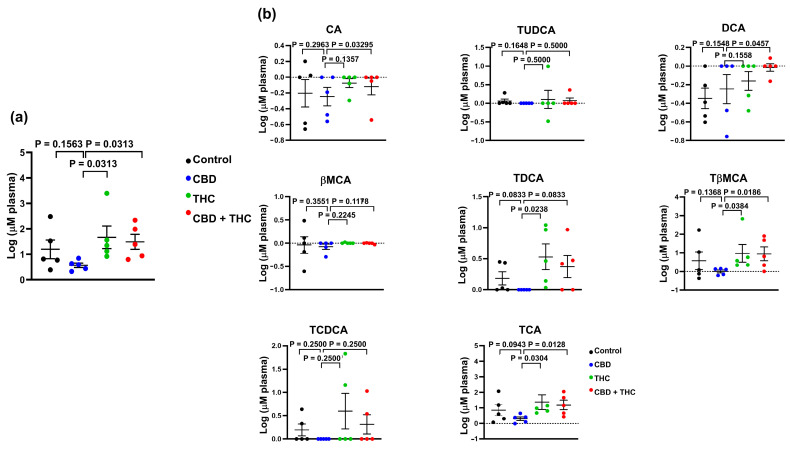
Bar plots showing the concentration of total bile acids (BA) (**a**)**,** and the indicated BA species (**b**) in plasma of cannabinoid treated KPC mice. The data were log-transformed and presented as mean ± SEM. The data analysis was performed using GraphPad Prism. The Mann–Whitney test was used to evaluate the differences in the BA profile within treatment groups. bMCA, β-muricholic acid; CA, cholic acid; DCA, deoxycholic; TCA, taurocholic acid; TDCA, taurodeoxycholic acid; TCDCA, taurochenodeoxycholic acid; TUDCA, tauroursodeoxycholic acid; TβMCA, tauro-β-muricholic acid.

**Figure 7:**
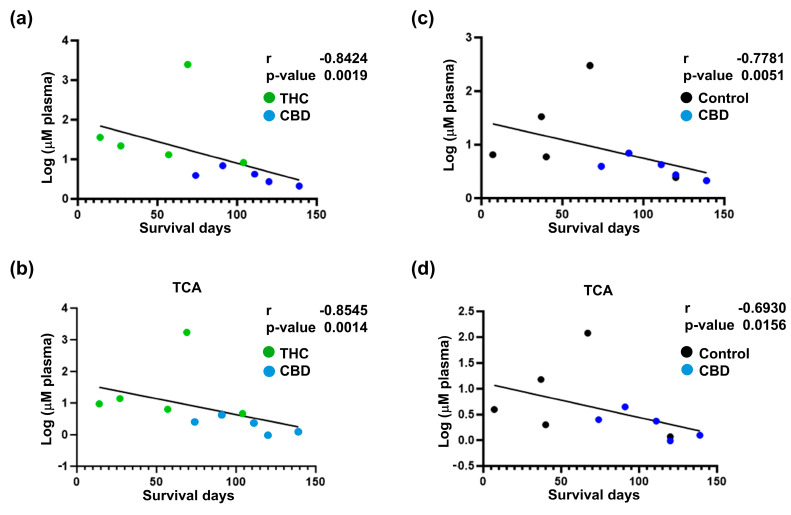
Correlation analysis between survival and circulatory bile acids (BA) profile for cannabinoid-treated mice. Correlation analysis for total circulatory BA profile (**a**,**c**) and circulatory taurocholic acid (TCA) (**b**,**d**) with survival. The results indicate a strong negative correlation between the decrease in total circulatory BA and TCA in the cannabidiol (CBD) group and improved survival outcomes. The log-transformed data is presented as scatter plots (including Spearman r and *p*-values) with simple linear regression. The data analysis was performed using GraphPad Prism.

To further evaluate the impact of cannabinoid therapy on the metabolism of BA, we evaluated the circulating levels of BA in this mice cohort. We observed that compared to treatment with other cannabinoids, CBD reduced the total concentration of BA in circulation (CBD versus THC, *p* = 0.0313; CBD versus THC + CBD, *p* = 0.0313) (Figure 6a). When looking at individual BA species, we observed that CBD-treated animals demonstrated lower levels of TCA (CBD versus THC, *p* = 0.0304; CBD versus THC + CBD, *p* = 0.0128), TβMCA (CBD versus THC, *p* = 0.0384; CBD versus THC + CBD, *p* = 0.0186), and TDCA (CBD versus THC, *p* = 0.0238) (Figure 6b). Interestingly, the total concentration of BA in blood showed a negative correlation with survival days (Figure 7a,c). Similar results were observed for individual BA, with TCA levels showing a strong association with survival days (Figure 7b,d and Supplementary Tables S2–S7).

## 3. Discussion

Due to its parallel incidence and mortality rates, PDAC is recognised as an aggressive and lethal malignancy. Despite advancements in diagnosis and treatment, PDAC survival rates have shown limited improvement [39]. Radical surgery offers a potentially cure but is feasible for only a small percentage of patients and is often limited by high recurrence rates. Consequently, chemotherapy either alone or in combination, becomes the primary treatment option for most PDAC patients [40]. Gemcitabine is the cornerstone of chemotherapy for advanced PDAC, but its efficacy is constrained by poor drug penetration and the development of chemoresistance [5,41]. This underscores an urgent need for novel therapeutic strategies to improve survival outcomes in PDAC.

A promising approach being actively explored involves combining novel intervention strategies with the current standard-of-care chemotherapy to target PDAC at multiple levels and improve survival outcomes. Among these strategies, cannabinoids, including phytocannabinoids, have garnered significant attention in PDAC research [12]. Notably, PDAC tissues exhibit elevated expression of cannabinoid receptors CB1, CB2, and GPR55 compared to normal pancreatic tissue [12,13,42]. This suggests that targeting the endocannabinoid system could offer therapeutic benefits for PDAC [41]. In this manuscript, the anticancer properties of two phytocannabinoids, CBD and THC, were investigated in PDAC. Phytocannabinoids have been widely studied for their anti-neoplastic effects, both alone and in combination with chemotherapy agents [12]. Our previous study demonstrated that CBD administration enhanced the efficacy of gemcitabine and improved survival outcomes in KPC mice. However, due to a small sample size, the survival improvement observed with CBD was not statistically significant [13]. In contrast, the present study utilised a larger cohort of KPC mice and found that CBD administration significantly improved survival rates.

The results of this study demonstrated that THC potentiated CBD in vitro, synergistically reducing PDAC cell viability. Similarly, Yang et al. reported that CBD + THC combination is more effective in PDAC and pancreatic stellate cells [43]. However, their study did not evaluate the strength of drug interactions in PDAC. Increased cytotoxicity of this combination has been documented in other cancers, including myeloma [44], non-small lung cancer [45] and breast cancer [46]. Interestingly, while our in vitro studies demonstrated clear synergistic cytotoxicity between CBD and THC, this effect did not translate into improved survival in vivo when the combination was tested in the KPC transgenic mouse model. Indeed, in contrast to these findings and the in vitro results, [43,47] i.p. administration of CBD + THC did not improve survival outcomes in KPC mice. Moreover, the data indicated that THC not only lacked activity as a monotherapy but also appeared to hinder CBD’s effects when used in combination. While the CBD-THC combination has shown efficacy in a PDAC xenograft model [47], the discrepancies in our data may reflect the complexity of the model used. This apparent discrepancy likely reflects fundamental differences between the simplified in vitro system and the complex in vivo tumour microenvironment. The KPC model is characterised by its desmoplastic stroma and intact immune system, features that are absent in standard two-dimensional cell culture. While in vitro assays primarily assess direct effects on tumour cell viability, in vivo outcomes are influenced by additional factors such as immune modulation, tumour–stroma interactions, drug pharmacokinetics, and tissue distribution. Notably, THC has been reported to exert immunosuppressive effects, suppressing T-cell proliferation and cytotoxic function, inhibiting IFN-γ and IL-2 production, shifting cytokine balance toward IL-10/TGF-β, and impairing anti-tumour immune responses [48], which may counteract CBD’s potential benefits in immune-competent in vivo settings. Rather than indicating pharmacological antagonism, we interpret the divergent results as context-dependent, shaped by the biological complexity of the host environment. These findings underscore the importance of integrating in vitro and in vivo models and suggest that the interaction between cannabinoids and the immune system warrants further mechanistic investigation. The KPC model, being an immunocompetent model and closely mimicking the histopathological features of human PDAC, provides a more accurate prediction of drug efficacy compared to xenograft models [49,50,51]. Despite the higher bioavailability expected with i.p. delivery, improved survival outcomes were not observed. This highlights the importance of using robust, clinically relevant models like KPC to evaluate therapeutic strategies in PDAC.

The THC + CBD combination was found to be effective in Capan-2 (PDAC cell line)-derived xenograft mouse models [47]. However, in this study, we investigated the anticancer properties of cannabinoids using a transgenic PDAC model, which differs from the xenograft models referenced above [47]. The increased complexity of the KPC model may explain the contrasting effects of CBD + THC observed in vivo compared to in vitro. As a genetically modified model, the KPC mouse exhibits both local and systemic responses to therapy, providing a more comprehensive representation of PDAC. Based on our findings, it was hypothesised that the observed improvement in the survival curve following CBD treatment could be attributed to drug effects occurring outside the primary tumour, highlighting the potential systemic mechanisms at play.

The dose of THC used in this study (10 mg/kg) was selected based on previous reports and preliminary assessments indicating it to be the highest dose tolerated by mice without inducing unacceptable behavioural side effects. At 15 mg/kg, mice exhibited overt psychotropic responses, including reduced locomotor activity, hypothermia, and behavioural suppression, which were not compatible with long-term survival studies or with ethical experimental endpoints. While 10 mg/kg is within the range reported to engage cannabinoid receptors and elicit biological activity, this dose may have been subtherapeutic in the context of pancreatic cancer. The lack of observed survival benefit with THC monotherapy may therefore reflect limited efficacy at this dose. Future studies incorporating full pharmacokinetic analyses and broader dose–response evaluations will be essential to determine the therapeutic potential and optimal dosing of THC in this setting.

The next step was to investigate whether i.p. administration of cannabinoids impacts microbial composition in the KPC model and whether this could explain the treatment-associated survival outcomes. Cannabinoid treatments resulted in marginal modulation of the gut microbiota, with small effect sizes and high inter-individual variation among KPC mice. Consistent alterations in gut microbiota associated with treatment were not observed, likely due to differing stages of disease progression caused by spontaneous tumour development in the model. Differential abundance analysis revealed that CBD-treated mice exhibited a reduction in opportunistic pathogens, such as *Salmonella* and *Pseudomonas,* alongside a modest increase in *Lactobacillus* and *Parasutterella*. *Lactobacillus plantarum* supernatant is known to enhance the chemosensitivity to fluorouracil in colon cancer cells [52] and plays a role in BA metabolism [53] through bile salt hydrolase enzymes that deconjugate primary BA [54]. Similarly, *Parasutterella* has been implicated in BA metabolism, particularly in deconjugation primary BA [55]. Cannabinoid treatments did not result in notable differences in faecal BA profile, likely due to the high variability in the KPC model. However, CBD treatment significantly reduced total circulating BA, particularly TCA, which correlated positively with survival outcomes, and TβMCA. These findings suggests that BA, rather than gut microbiota, could serve as dynamic biomarkers in PDAC. Given the importance of both TCA and TβMCA levels in modulating the metabolism of BA in mice [30], these results suggest that CBD might negatively modulate the metabolism of BA. Interestingly, previous studies have identified TCA as a potential diagnostic marker in PDAC patients [56]. While the role of BA in pancreatitis and pancreatic injury is established [31,57], their influence on PDAC progression remains unclear. An intriguing finding in our study is the inverse correlation between TCA and TβMCA, and survival in CBD-treated mice. While this association is robust, the current data do not allow us to determine whether BA modulation is a mechanistic driver of CBD’s therapeutic effects or a downstream consequence of broader tumour or metabolic changes. One possibility is that CBD acts upstream, modulating bile acid synthesis, transport, or clearance directly or indirectly, thereby contributing to improved outcomes. Alternatively, the reduction in bile acid levels may reflect secondary changes in tumour progression or host metabolism induced by CBD treatment

Emerging literature suggests that cannabinoids, including CBD, can interact with nuclear receptors such as PPARγ and GPR55, which are functionally linked to bile acid metabolism. Furthermore, nuclear receptors such as FXR, PXR, CAR, and VDR play key roles in bile acid homeostasis and may serve as mechanistic intermediaries. Notably, TβMCA is an established antagonist of FXR, a receptor increasingly recognised for its role in metabolic regulation and pancreatic cancer biology, highlighting a potentially relevant mechanistic axis in our study [30]. These findings raise the hypothesis that the CBD-induced reduction in conjugated bile acids such as TβMCA may relieve FXR inhibition, thereby altering transcriptional programs relevant to tumour progression and metabolic homeostasis. Although speculative at this stage, this bile acid–nuclear receptor axis offers a compelling mechanistic framework that warrants further investigation. Future studies integrating targeted receptor analysis, transcriptomics, and pharmacological modulation of bile acid pathways will be critical to disentangle causality and define whether bile acid changes mediate or merely accompany the therapeutic effects of CBD.

The concentrations of CBD and THC used in this study were 100 mg/kg and 10 mg/kg, respectively. These doses were selected based on previous preclinical studies investigating the anti-neoplastic properties of cannabinoids [13]. While CBD has demonstrated a favourable safety profile in both preclinical and clinical settings, THC’s psychoactive effects and potential toxicity necessitate careful consideration. Notably, the doses used in this study are within the range previously reported to exert anticancer effects without inducing significant toxicity in murine models. However, potential adverse effects such as immunosuppression or metabolic alterations must be accounted for in future studies.

Although we did not directly quantify plasma levels of CBD or THC in this study, we considered existing pharmacokinetic data to relate our in vitro findings to the in vivo outcomes. Intraperitoneal administration is commonly used in preclinical cancer models due to its reliable systemic absorption and prolonged exposure in the peritoneal cavity. Previous studies have shown that a single i.p. dose of CBD at 100 mg/kg yields peak plasma concentrations in the low micromolar range, consistent with the IC_50_ values observed in our in vitro assays [13]. Similarly, THC at 10 mg/kg has been reported to reach plasma levels sufficient to engage cannabinoid receptors without causing overt toxicity. These data suggest that the doses used in our study likely achieved biologically relevant systemic concentrations. However, we acknowledge that individual differences in drug metabolism, tumour burden, and tissue distribution may influence actual exposure levels in vivo. The lack of direct pharmacokinetic analysis is a limitation of our study and highlights the need for future investigations integrating pharmacokinetics with therapeutic efficacy.

For potential clinical translation, the administered doses should be evaluated in the context of human-equivalent dosing. Standard allometric scaling from murine to human models suggests that the doses used in this study correspond to approximately 8.1 mg/kg for CBD and 0.81 mg/kg for THC in humans. Given that CBD is well tolerated in clinical settings at doses up to 20 mg/kg/day, these findings support its potential therapeutic application in PDAC. Conversely, THC’s psychoactive properties and associated side effects may limit its clinical utility, necessitating dose optimisation strategies to balance efficacy and tolerability. Further pharmacokinetic and toxicological studies are required to establish the optimal dosing regimen for cannabinoids in human PDAC patients. Therefore, these findings reinforce the potential of CBD as a therapeutic candidate for PDAC, warranting further clinical investigation to assess its safety, efficacy, and mechanistic impact in human patients. The clinical significance of the in vivo size of the effect observed in this study is noteworthy. PDAC remains a devastating malignancy with limited therapeutic options and dismal survival outcomes. The ability of CBD to significantly improve survival in the KPC model highlights its potential translational relevance. Unlike xenograft models, the KPC model closely mimics the genetic, histopathological, and immune landscape of human PDAC, offering a more accurate prediction of clinical efficacy. The improvement in survival outcomes observed in this study, particularly when other treatment combinations, such as CBD + THC, failed to yield similar results, underscores the systemic and multifaceted action of CBD. These findings suggest that CBD could provide therapeutic benefits beyond tumour-localised effects, potentially influencing systemic disease progression and secondary complications, as indicated by the observed alterations in bile acid metabolism. Such effects may offer new avenues for improving the quality of life and extending survival in PDAC patients, underscoring the importance of further clinical evaluation of CBD as a complementary therapeutic strategy in PDAC treatment. While our study demonstrates a significant survival benefit in CBD-treated KPC mice, we acknowledge the absence of molecular or histological endpoints directly confirming anti-tumour activity in vivo. In this spontaneous tumour model, where lesion onset and progression vary considerably among animals, standardised collection of tumour tissue at equivalent stages was not feasible. Consequently, detailed immunohistochemical analyses (e.g., Ki-67, cleaved caspase-3) or transcript-level profiling were not incorporated in the current study design. We did perform H&E staining to confirm tumour presence and assess tissue architecture, but more specific mechanistic markers were outside the scope of this survival-focused investigation.

Nevertheless, the use of survival as a primary endpoint in the KPC model, widely regarded as one of the most clinically relevant and aggressive models of PDAC, provides robust translational value. Moreover, in our previous work using this same model, we demonstrated that CBD treatment reduced Ki-67 and phospho-ERK expression in pancreatic tumours [13], supporting its capacity to inhibit tumour cell proliferation. These findings reinforce the biological relevance of the survival benefit observed in the present study. Future work will be directed at incorporating longitudinal imaging and timepoint-specific tissue analyses, including IHC and RNA profiling, to further elucidate the mechanisms underpinning the observed therapeutic effects.

## 4. Materials and Methods

### 4.1. Animal Studies

C57BL/6J Cre (C57) and KRASWT/G12D/TP53WT/R172H/Pdx1-Cre+/+ (KPC) were obtained from the Animal Research Centre (ARC) in Murdoch, Western Australia. All animal procedures were performed under strict adherence to the Australian Code for the Care and Use of Animals for Scientific Purposes and were approved by the Curtin University Animal Ethics Committee (Approval number 2019-22). Mice were maintained in ventilated cages with food and water in standard conditions (12 h light/dark cycle, 21 °C, 60% humidity and 20 air changes/h). The spontaneous development of pancreatic tumours in KPC mice was assessed by palpation. Once the tumour reached a palpable size, the animals were administered i.p. treatments (CBD 100 mg/kg, THC 10 mg/kg, CBD 100 mg/kg + THC 10 mg/kg, and vehicle control) once daily until the treatment endpoint (death or sacrifice). Over this time, mice were monitored daily (palpated and weighted). The daily i.p. injection treatment was prepared in DMSO, 0.9% NaCl and Tween 80 for in vivo experiments. All treatment groups, including controls, received matched vehicle injections, and injection volumes were standardised across animals to ensure comparability. Injection volumes were nominally adjusted based on body weight; however, due to minimal variability between animals, the final volumes were effectively uniform across experimental groups. The animals were sacrificed using isoflurane for anaesthesia followed by cervical dislocation when showing signs of discomfort/pain (assessed using the Grimace scale), substantial weight loss (more than 20% of initial weight), cachexia, weakness, and abdominal ascites. Blood (cardiac puncture), tissues (pancreas, liver, lungs, and spleen) and caecal content were collected from the mice when sacrificed. While the collected faeces, caecal contents, and tissues were snap-frozen, the blood samples were centrifuged at 17,000× *g* for 5 min, and the collected plasma was stored at −80 °C along with other samples. There were no observed side/adverse effects of i.p. cannabinoid treatments at the given concentrations. For immunohistochemistry (IHC), samples were collected in formalin, dehydrated and paraffin embedded. The experiment’s group sizes were initially designed to be equal. However, some samples were ultimately lost due to strain-related secondary illnesses in the KPC mice. These mice are predisposed to developing off-target conditions, which include benign lymphomas (notably in the mesenteric and inguinal lymph nodes) and lipomas characterised by uncontrolled cell growth, particularly on the face. Additionally, external vulvar papilloma was observed, further impacting the health of the mice. In such cases, mice exhibiting severe symptoms were humanely euthanized to prioritise animal welfare, in compliance with ethical research guidelines. This accounted for the uneven final group sizes. All animal experiments were conducted using randomization to assign subjects to experimental groups, ensuring unbiased distribution. Investigators were blinded to treatment allocation during data collection and analysis to minimise potential bias.

### 4.2. Immunohistochemistry

Paraffin-embedded KPC and C57 pancreatic tissues were sliced into 4 µm sections using a microtome. The slides were dewaxed and rehydrated by incubation in xylene, followed by ethanol (100%, 70%, and 50%) and water for 5 min each. Sodium citrate buffer (10 mM sodium citrate, 0.05% Tween 20, pH 6.0) was used for heat-induced antigen retrieval. After staining with haematoxylin and eosin, the sections were washed and cleared using xylene. The tissue sections were washed with water between the haematoxylin and eosin staining. Mounting medium Entellan (Sigma, St. Louis, MO, USA) was used, and the slides were visualised under a light microscope.

### 4.3. Quantitative Profiling of Bile Acids

Bile acids were extracted from ~80 mg of faeces/caecal content using our previously described method [58]. Briefly, faecal pellets were homogenised using 0.1 mm diameter zirconia/silica beads in acetonitrile. Following centrifugation at 17,000× *g* for 15 min 4 °C, the supernatant was removed, dried under a nitrogen stream, and reconstituted in 90% acetonitrile in water. Plasma BA were extracted using liquid–liquid extraction with acetonitrile and processed as faecal pellets but omitting the homogenisation step. For BA profiling, reconstituted samples were subjected to chromatographic separation using Ultra-High-Performance Liquid Chromatography (UHPLC Agilent 1260, Santa Clara, CA, USA) and detection using LC-MS (Agilent QTOF 6540) as described [58,59]. For quantification, plasma samples were spiked with a mix of deuterated internal standards 5β-cholanic acid-3α,12α-diol-2,2,4,4-d4 (CA-D4, C1070-015 Steraloids, Newport, RI, USA), 5β-cholanic acid-3α,12α-diol-2,2,4,4-d4 (DCA-D4, C1070-015 Steraloids), and 5β-cholanic acid-3α,7α-diol-2,2,4,4-d4 (CDCA-D4, C0940-015 Steraloids) and one analogous bile acid 23-Nor-5β-cholanic acid-3α, 7α, 12α triol (23-NCA, N2450-000 Steraloids), prior to extraction. The area of the internal standard was used for quantifying the different BA species as we previously described [59]. The following BA species were used to generate a reference library of mass spectral-retention time: β-muricholic acid (Santa Cruz, sc477731, Dallas, TX, USA), α-muricholic acid (Steraloids, C1890-000), ω-muricholic acid (Steraloids, C1888-000), tauro-β-muricholic acid (Steraloids, C1899-000), taurocholic acid (Santa Cruz, sc220189), taurolithocholic acid (Cayman Chemicals, 17275, Ann Arbor, MI, USA), taurochenodeoxycholic acid (Steraloids, C0990-000), taurodeoxycholic acid (Steraloids, C1162-000), tauroursodeoxycholic acid (Steraloids, C1052-000), glycolithocholic acid (Steraloids, C1435-000), glycochenodeoxycholic acid (Steraloids, C0962-000), glycoursodeoxycholic acid (Steraloids, C1025-000), glycocholic acid (Steraloids, C1927-000), glycodeoxycholic acid (Steraloids, C1087-000), cholic acid 7-sulphate (Cayman Chemicals, 9002532), cholic acid (Sigma, C1129), deoxycholic acid (Sigma, D2510), lithocholic acid (Sigma, L6250), chenodeoxycholic acid (Sigma, C1050000), ursodeoxycholic acid (Steraloids, C1020-000).

### 4.4. Bacterial DNA Isolation and Profiling of the Faecal-Associated Bacterial Biota

DNA was isolated from ~80 mg of homogenised stool using the QIAamp Fast DNA Stool kit (QIAGEN, Hilden, Germany), as previously described [58]. The bacterial component of the faecal microbiota was profiled using an amplicon sequencing approach targeting the V3-V4 region of the 16S rRNA gene. Polymerase chain reaction libraries were generated at Genewiz (South Plainfield, NJ, USA) and sequenced using V3 chemistry (Illumina, San Diego, CA, USA) in a 2 × 300 bp run using a Illumina MiSeq (Illumina, Inc., San Diego, CA, USA).

The generated 4,311,464 sequences were distributed in 20 samples and one negative extraction controls, with a good quality score (>92% of reads with an average PhreD score higher or equal to 30). Quality-based processing and joint of paired-end reads were performed as previously reported [60]. This quality-based filtering process resulted in 1,602,103 high-quality (>94% sequences with a Phred score higher or equal to 35) paired-end sequences (average length 398 bp). Taxonomy assignment was performed using the SILVAngs pipeline [61,62] and the last release of the SILVA SSU taxonomy reference (release 138.1). The differential abundance analysis was performed using the Analysis of Compositions of Microbiomes with Bias Correction (ANCOM-BC) pipeline [38].

### 4.5. Cell Culture

AsPC-1 (CRL-1682™) and HPAF-II (CRL-1997) were obtained from the American Type Culture Collection panel of PDAC cell lines (TCP-1026™). RPMI-1640 (Sigma Cat#. R8758) and MEM Eagle’s medium (Sigma Cat# M5650) were used to culture AsPC-1 and HPAF-II, respectively, supplemented with 10% FBS, 10 nM glutamine and 1% penicillin and streptomycin (PS) of the final concentration. Cells at passages 3–7 were used for in vitro experiments.

### 4.6. Cell Viability Assay

3-(4,5-dimethylthiozol-2-yl) 2,5-diphenyltetrazolium bromide solution (MTT, Sigma) diluted in phosphate-buffered saline (PBS, 5 mg/mL) was used to evaluate the cell viability of the treated PDAC cell lines and CAFs. Cell viability experiments were conducted in a 96-well plate, where the cells were treated with compounds added in triplicates for 72 h and then incubated with MTT solution (10 µL MTT with 90 µL serum-free medium) for 1–4 h until they appeared blue when checked under the microscope. After the colour change, MTT was removed, and the plates were dried for 2 h. Following this, 70 µL/well DMSO was added, and the absorbance was measured between 520 and 600 nm using Kaliedo^TM^ software v3.0 of the EnSight^®^ multimode plate reader (PerkinElmer, VIC, Australia). Raw data were analysed using Excel and presented as the mean (percentage of viability) ± SEM of three independent experiments normalised to control.

To assess cell viability following cannabinoid treatments, AsPC-1 and HPAF-II were seeded at 5000 cells/well in a 96-well plate and left undisturbed for 24 h. The CBD and THC treatments were added at 0.25 µg/mL, 0.5 µg/mL, 1 µg/mL, 2 µg/mL, 2.5 µg/mL, 5 µg/mL, 7.5 µg/mL, 10 µg/mL, 15 µg/mL, 20 µg/mL final concentration. In addition, the cells were treated with CBD (0.25 µg/mL, 0.5 µg/mL, 1 µg/mL, 2 µg/mL, 2.5 µg/mL, 5 µg/mL, 7.5 µg/mL, 10 µg/mL, 15 µg/mL, 20 µg/mL) in combination with THC 2.5 µg/mL and 7.5 µg/mL for 72 h. The absorbance was recorded following MTT addition after 72 h. The strength of drug interactions between CBD and THC was quantitated using the Chou-Talay method on CompuSyn software (Biosoft, San Francisco, CA, USA). The Chou-Talay method based on the median-effect equation provides a theoretical base for the combination index (CI)—isobologram equation. CI < 1, CI > 1 and CI = 1 indicated synergism, antagonism, and additive effects, respectively [63,64].

CBD and THC (THC Pharm, Frankfurt, Germany) were dissolved in DMSO to prepare 100 mM stock solutions. The stock solutions were further diluted in growth medium for in vitro treatments ensuring a final DMSO concentration below 0.5% to avoid cytotoxicity.

### 4.7. Statistical Analysis

Cell culture analyses in Figure 1, Kaplan–Meier survival curves in Figure 2, and correlation analyses in Figure 7 were generated using the GraphPad PRISM V10.0 software. Cell culture data was obtained from a minimum of three independent experiments using GraphPad PRISM V10.0 software. The statistical tests employed are indicated in the figure legend and the selection was based on the nature of the data and the hypotheses being tested. Microbiome and bile acid analyses were performed in R statistical software (v4.3.0) within the RStudio environment (v2023.03.0). We used vegan (v2.6-4) to calculate the Shannon alpha diversity index and for conducting permutational multivariate analysis of the variance (PERMANOVA). Differentially abundance analysis was performed using the ANCOMBC R package (v2.2.0). When multiple hypotheses were tested simultaneously, we adjusted the *p*-values for multiple comparisons by controlling the family-wise error rate using the Dunnett’s post hoc test, or the false discovery rate method based on the nature and dimensionality of the data. The cut-off for statistical significance was set at *p* < 0.05.

## 5. Conclusions

Overall, our study highlights that cannabinoids can induce significant alterations in the gut microbiota-BA axis in KPC models. The CBD-driven changes in BA metabolism and gut microbiota composition were associated with improved survival, underscoring their functional relevance. Importantly, our findings support the use of the BA–microbiota axis as a dynamic biomarker of therapeutic response to CBD in PDAC, offering a novel avenue for both mechanistic understanding and clinical monitoring.

## Data Availability

Data will be available upon request and further inquiries can be directed to the corresponding author. The sequencing data has been deposited in the Sequencing Read Archive under the BioProject accession number PRJNA1211174.

## Supplementary Materials

The following supporting information can be downloaded at: https://www.mdpi.com/article/10.3390/ijms26167733/s1.

## Abbreviations

The following abbreviations are used in this manuscript:

BA	Bile acids
CBD	Cannabidiol
CI	Combination index
IHC	Immunohistochemistry
i.p.	Intraperitoneal
KPC	KRASWT/G12D/TP53WT/R172H/Pdx1-Cre+/+
MTT	3-(4,5-dimethylthiazol-2-yl)-2,5-diphenyl-2H-tetrazolium bromide
OTU	Operational taxonomic unit
PDAC	Pancreatic ductal adenocarcinoma
THC	Delta-9-tetrahydrocannabinol
