# Supplementary material for: Cannabidiol Is Associated with Improved Survival in Pancreatic Cancer and Modulation of Bile Acids and Gut Microbiota
Source: Int J Mol Sci. 2025 Aug 10;26(16):7733. doi: 10.3390/ijms26167733 (PMC12386833; doi:10.3390/ijms26167733)
Supplement: Supplementary file 1 [file ijms-26-07733-s001.zip › ijms-3723978-supplementary.pdf]

## Supplementary Materials

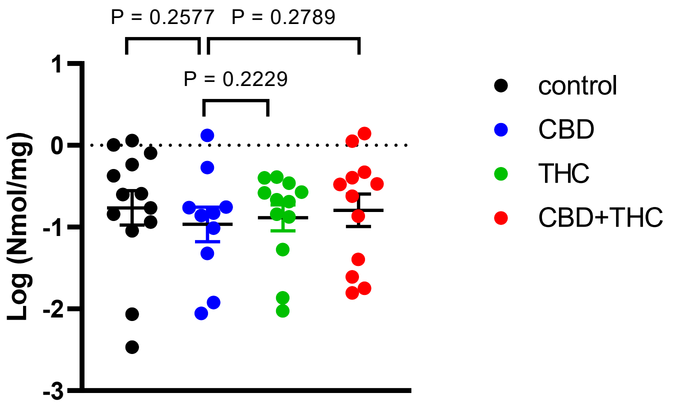


**Supplementary Figure S1.** Bar plots showing the concentration of total BAs in faeces of cannabinoid treated KPC mice. The data were log-transformed and presented as mean ± SEM. The data analysis was performed using GraphPad Prism. The Mann-Whitney test was used to evaluate the differences in the BAs profile within treatment groups.

**Supplementary Table S1.** Descriptive statistics of KPC mice treated with cannabinoids.

| Parameters | Treatments | | | |
|---|---|---|---|---|
| | Control | CBD | THC | CBD + THC |
| Number of animals per group | 18 | 16 | 16 | 17 |
| 25% percentile | 25 | 44.25 | 28.75 | 22.00 |
| Median | 47.00 | 82.50 | 60.00 | 45.00 |
| 75% percentile | 71.50 | 117.8 | 88.50 | 85.50 |
| Mean | 50.72 | 83.56 | 58.19 | 56.53 |

**Supplementary Table S2.** Correlation between circulating bile acids levels and survival days in control and THC-treated KPC mice

| Bile acid | Spearman r | P-value |
|---|---|---|
| TCA | -0.06667 | 0.4326 |
| CA | -0.1563 | 0.3335 |
| TUDCA | 0.3755 | 0.1437 |
| DCA | 0.7254 | 0.0229* |
| βMCA | -0.006465 | 0.5000 |
| TDCA | -0.3497 | 0.1616 |
| TωMCA | 0.04242 | 0.4592 |
| TCDCA | -0.02048 | 0.4810 |
| Total bile acids | -0.1030 | 0.3925 |

**Supplementary Table S3.** Correlation between circulating BAs levels and survival days in control and CBD-treated KPC mice

| Bile acid | Spearman r | P-value |
|---|---|---|
| TCA | -0.6930 | 0.0156* |
| CA | -0.2523 | 0.2400 |
| TUDCA | -0.4165 | 0.12222 |
| DCA | 0.1944 | 0.2914 |
| βMCA | -0.3199 | 0.1819 |
| TDCA | -0.6956 | 0.0153* |
| TωMCA | -0.3830 | 0.1365 |
| TCDCA | -0.4165 | 0.1222 |
| Total bile acids | -0.7781 | 0.0054* |

**Supplementary Table S4.** Correlation between circulating BAs levels and survival days in CBD and THC-treated KPC mice

| Bile acid | Spearman r | P-value |
|---|---|---|
| TCA | -0.8545 | 0.0014* |
| CA | -0.2909 | 0.2072 |
| TUDCA | 0.2335 | 0.3111 |
| DCA | -0.07511 | 0.4190 |
| βMCA | -0.3356 | 0.1806 |
| TDCA | -0.6623 | 0.0212* |
| TωMCA | -0.8424 | 0.0019* |
| TCDCA | -0.4930 | 0.0889 |
| Total bile acids | -0.8424 | 0.0019* |

**Supplementary Table S5.** Correlation between circulating BAs levels and survival days in control and CBD+THC-treated KPC mice

| Bile acid | Spearman r | P-value |
|---|---|---|
| TCA | 0.006061 | 0.5000 |
| CA | -0.3439 | 0.1653 |
| TUDCA | 0.4250 | 0.1139 |
| DCA | 0.5878 | 0.0390* |
| βMCA | -0.1313 | 0.3606 |
| TDCA | 0.08129 | 0.4124 |
| TωMCA | 0.05455 | 0.4458 |
| TCDCA | 0.2253 | 0.2647 |
| Total bile acids | 0.006061 | 0.5000 |

**Supplementary Table S6.** Correlation between circulating BAs levels and survival days in CBD and CBD+THC-treated KPC mice

| Bile acid | Spearman r | P-value |
|---|---|---|
| TCA | -0.4303 | 0.1091 |
| CA | -0.5625 | 0.0481* |
| TUDCA | 0.4602 | 0.2000 |
| DCA | -0.09559 | 0.3992 |
| βMCA | -0.2595 | 0.2377 |
| TDCA | 0.5000 | 0.5000 |
| TωMCA | -0.3818 | 0.1394 |
| TCDCA | -0.01730 | 0.4889 |
| Total bile acids | -0.4182 | 0.1163 |

**Supplementary Table S7.** Correlation between circulating Bas levels and survival days in all the treatments (control, CBD, THC and CBD+THC)

| Bile acid | Spearman r | P-value |
|---|---|---|
| TCA | -0.3949 | 0.0424* |
| CA | -0.4061 | 0.0378* |
| TUDCA | 0.2333 | 0.1611 |
| DCA | 0.3440 | 0.0688 |
| βMCA | 0.1240 | -0.2709 |
| TDCA | -0.3399 | 0.0713 |
| TωMCA | -0.2813 | 0.1148 |
| TCDCA | -0.1938 | 0.2065 |
| Total bile acids | -0.4325 | 0.0284* |
